# Increased Glutamate Receptor and Transporter Expression in the Cerebral Cortex and Striatum of *Gcdh* ^-/-^ Mice: Possible Implications for the Neuropathology of Glutaric Acidemia Type I

**DOI:** 10.1371/journal.pone.0090477

**Published:** 2014-03-04

**Authors:** Valeska Lizzi Lagranha, Ursula Matte, Talita Giacomet de Carvalho, Bianca Seminotti, Carolina Coffi Pereira, David M. Koeller, Michael Woontner, Stephen I. Goodman, Diogo Onofre Gomes de Souza, Moacir Wajner

**Affiliations:** 1 Departamento de Bioquímica, Instituto de Ciências Básicas da Saúde, Universidade Federal do Rio Grande do Sul, Porto Alegre, Rio Grande do Sul, Brazil; 2 Centro de Terapia Gênica, Centro de Pesquisas Experimental, Hospital de Clínicas de Porto Alegre, Porto Alegre, Rio Grande do Sul, Brazil; 3 Departments of Pediatrics, Molecular and Medical Genetics, Oregon Health & Science University, Portland, Oregon, United States of America; 4 School of Medicine, University of Colorado at Denver, Aurora, Colorado, United States of America; 5 Serviço de Genética Médica, Hospital de Clínicas de Porto Alegre, Porto Alegre, Rio Grande do Sul, Brazil; University of Iowa, United States of America

## Abstract

We determined mRNA expression of the ionotropic glutamate receptors NMDA (NR1, NR2A and NR2B subunits), AMPA (GluR2 subunit) and kainate (GluR6 subunit), as well as of the glutamate transporters GLAST and GLT1 in cerebral cortex and striatum of wild type (WT) and glutaryl-CoA dehydrogenase deficient (*Gchh* ^-/-^) mice aged 7, 30 and 60 days. The protein expression levels of some of these membrane proteins were also measured. Overexpression of NR2A and NR2B in striatum and of GluR2 and GluR6 in cerebral cortex was observed in 7-day-old *Gcdh* ^-/-^. There was also an increase of mRNA expression of all NMDA subunits in cerebral cortex and of NR2A and NR2B in striatum of 30-day-old *Gcdh* ^-/-^ mice. At 60 days of life, all ionotropic receptors were overexpressed in cerebral cortex and striatum of *Gcdh* ^-/-^ mice. Higher expression of GLAST and GLT1 transporters was also verified in cerebral cortex and striatum of *Gcdh* ^-/-^ mice aged 30 and 60 days, whereas at 7 days of life GLAST was overexpressed only in striatum from this mutant mice. Furthermore, high lysine intake induced mRNA overexpression of NR2A, NR2B and GLAST transcripts in striatum, as well as of GluR2 and GluR6 in both striatum and cerebral cortex of *Gcdh* ^-/-^ mice. Finally, we found that the protein expression of NR2A, NR2B, GLT1 and GLAST were significantly greater in cerebral cortex of *Gcdh* ^-/-^ mice, whereas NR2B and GLT1 was similarly enhanced in striatum, implying that these transcripts were translated into their products. These results provide evidence that glutamate receptor and transporter expression is higher in *Gcdh* ^-/-^ mice and that these alterations may be involved in the pathophysiology of GA I and possibly explain, at least in part, the vulnerability of striatum and cerebral cortex to injury in patients affected by GA I.

## Introduction

Glutaric acidemia type I (GA I, McKusick 23167; OMIM # 231670) is an autosomal recessive inherited neurometabolic disease caused by deficiency of glutaryl-CoA dehydrogenase activity (GCDH, EC 1.3.99.7), with an estimated incidence of 1∶30,000-1∶100,000 live-births, reaching a much higher prevalence in some communities (1∶300) [1]–[5]. Increased concentrations of glutaric acid (GA, 500–5000 µmol/l) and 3-hydroxyglutaric acid (3-HGA), at lower amounts (40–200 µmol/l), are found in the body fluids and in the brain of GA I patients [1], [2], [6]–[8]. GA-I is considered a “cerebral” organic aciduria because affected patients present essentially neurological symptoms including diskenesia/dystonia and spasticity that appear especially after encephalopathic crises, which occur between 6 and 36 months of age and are accompanied by bilateral destruction of caudate and putamen [9], [10]. Macrocephaly and frontotemporal atrophy is a distinctive radiological appearance frequently detected at birth [7], [10]. Cranial MRI findings usually show a pattern of progressive spongiform white matter changes with cortical hypoplasia (leukoencephalopathy) and degeneration of the basal ganglia, whereas histopathological studies reveal vacuolization, subdural hemorrhages with loss of medium spiny neurons, as well as astrogliosis [1], [6], [10]–[14].

At present, the exact pathomechanisms of the neurological symptoms and brain abnormalities of the affected patients are still obscure. However, there are *in vitro* and *in vivo* experimental evidences indicating that disruption of mitochondrial energy metabolism [15]–[19], oxidative stress [20]–[26] and glutamatergic excitotoxicity [27]–[39] are involved in the pathogenesis of this disorder.

The *post mortem* examination of the basal ganglia and cerebral cortex of patients with GA I revealing postsynaptic vacuolization characteristic of glutamate-mediated brain injury [1] and the structural similarity between glutamate and the main organic acids (GA and 3-HGA) accumulated in GA I support a possible role of excitotoxicity as a relevant mechanism underlying the neurotoxicity in GA-I. However, other experimental observations did not confirm this pathomechanism [39], [40], implying that the importance of excitotoxicity is still controversial.

Glutamate is the main excitatory neurotransmitter in the mammalian brain and its interactions with specific membrane receptors are responsible for many CNS functions such as cognition, memory and movement [41]. The role of glutamate in mammalian brain is mediated by activation of glutamate-gated cation channels termed ionotropic glutamate receptors (iGLUR) and of GTP-binding protein (G-protein)-linked receptors termed metabotropic receptors (mGLUR) [42], [43]. iGLUR can be divided into N-methyl-D-aspartate (NMDA: NR1, NR2A-D and NR3A-B subunits) and non-NMDA, the latter including the a-amino-3-hydroxy-5-methyl-4-isoxazole propionic acid (AMPA: GluR1-4 subunits) and kainate (GluR5-7 and KA1-2 subunits) receptors. mGLUR have been divided into groups I, II and III [41]–[44].

Glutamate receptors (GLUR) are involved in a variety of physiological processes during brain development, including synaptogenesis and synaptic plasticity, and present a unique pattern of susceptibility to toxicity mediated by differential activation of the receptor subtypes [45]. These receptors are ligand-gated ion channels permeable to Na^+^, K^+^ and Ca^2+^ that mediate the fast depolarization of the post-synaptic membrane after glutamatergic stimuli and induce post-synaptic action potentials to propagate neuronal information [46].

Regarding the iGLUR, NMDA expression reaches the highest level in hippocampus and neocortex in the first postnatal week in the rat, whereas AMPA receptors density peaks in these structures take place in the second postnatal week [47], [48]. In contrast, in the neonatal rat striatum NMDA receptor maturation occurs later than kainate and AMPA receptor expression [49], [50].

The synaptic actions of glutamate are terminated by its removal from the synaptic cleft by a high-affinity sodium-dependent excitatory amino acid transporter (EAAT) system, mainly located in the astrocytic membranes [41], [51], [52]. These astroglial glutamate transporters are called GLAST (EAAT1) and GLT1 (EAAT2) [53]. In the rat, GLAST is expressed at birth, whereas GLT1 is mainly expressed during the second to third postnatal week. Both transporters are fully expressed by postnatal week 5, but GLT1 is the predominant glutamate astroglial transporter in the adult brain [54].

Overstimulation of GLUR leads to excessive influx of Ca^2+^ and Na^+^ that activate lipases, proteases, phosphatases and other enzymes that may lead to neuronal death, a process called primary excitotoxicity that is related to the neuropathology of common acute and chronic brain disorders [55]–[61]. Secondary excitotoxicity usually caused by disruption of cellular energy homeostasis may lead to a reduction of glutamate uptake by astrocytes resulting in increased amounts of glutamate in the synaptic cleft that overstimulates GLUR in a vicious circle [41], [62].

A knockout (KO) GA I model was developed in mice by replacing the glutaryl-CoA dehydrogenase gene with an in-frame beta-galactosidase cassette to mimic the *in vivo* human condition in order to allow investigation on the pathomechanisms causing the brain damage of glutaric acidemic patients [63]. The KO mice (*Gcdh* ^-/-^) present increased cerebral, blood and urine GA and 3-HGA levels and displayed vacuolization in the frontal cortex. However, the animals did not develop striatal damage typical of the human disease even when submitted to metabolic or infectious stress. This model was later improved by exposing these mice to high protein or lysine (Lys) intake, which provoked neuronal loss, myelin disruption and gliosis mostly in the striatum and deep cortex [64], [65]. Oral Lys overload to weaning (4-week-old) *Gcdh* ^-/-^ mice resulted in a predominant increase of brain Lys and GA concentration after 48 h of Lys exposure. Cortical and striatal alterations paralleled with a simultaneous decrease of Lys and increase of brain GA levels were also seen, indicating GA formation from Lys. These investigators suggested that the brain lesions observed histopathologically in the *Gcdh* ^-/-^ animals submitted to Lys overload were probably due to the increase of brain GA concentration [65].

Despite the intense investigation of the neuropathology of GA I, the mechanisms underlying the window of vulnerability of acute striatum (3–36 months of age) and chronically progressive cortical damage affecting GA I patients are not yet fully established. In this scenario, previous reports suggest that a variable expression pattern of GLUR may result in a regional- and age-specific window of susceptibility in various neuropathological states [66], [67]. We therefore postulate that a variable expression of distinct GLURs and transporters along development in striatum and cerebral cortex of *Gcdh^-/-^* mice may potentially provide a “window of vulnerability” of these structures to neurotoxins such as GA and 3-HGA. Indeed, a previous report showing differential age and brain structural related effects of GA and 3-HGA on glutamate binding to GLURs and transporters during rat brain development may support this hypothesis [32].

Therefore, in the present study we aimed to determine and compare the mRNA expression of ionotropic glutamate receptor subunits and transporter subtypes in the cerebral cortex and striatum from wild type (WT) and *Gcdh* ^-/-^ mice at distinct periods of postnatal development (7, 30 and 60 postnatal day). We employed quantitative real-time PCR (RT-qPCR) to examine the expression levels of 7 genes that encode the NMDA subunits NR1, NR2A and NR2B, the AMPA subunit GluR2 and the kainate subunit GluR6, as well as of the genes that encode the astrocytic transporters GLAST and GLT1. We also investigated the effect of a high Lys diet (4.7%) on the mRNA expression of these subunits in 30-day- old normal (WT) and *Gcdh* ^-/-^ mice. Finally, we measured the expression of some of these proteins by western blotting in both cerebral structures from WT and *Gcdh* ^-/-^ mice at 60 days of postnatal life.

## Materials and Methods

### Animals and brain preparation

Female and male mice knockout for the gene *Gcdh* (*Gcdh* ^-/-^) on a 129SvEv/background (kindly donated by Prof. Stephen Irwin Goodman, University of Colorado, EUA) and wild type (*Gcdh* ^+/+^, WT) mice of the same strain were used. The animals were maintained at Animal Experimentation Unit at the Research Center of Hospital de Clínicas de Porto Alegre, fed *ad libitum* with standard chow and maintained under a constant photoperiod of light/dark 12:12 h with controlled temperature (22±1°C) and humidity (50±10%).

Animals (*Gcdh* ^-/-^ and WT) at 7, 30 and 60 days of age were used (n = 5 animals per group). All were sacrificed by decapitation without anaesthesia. The brain was rapidly removed and placed on a Petri dish on ice. The olfactory bulb, pons, medulla and cerebellum were discarded and the cerebral cortex and striatum dissected. These structures were homogenized in 1 mL PBS 1x and total RNA was extracted as described below. For protein expression, these structures were homogenized in 50 mM Tris–Cl buffer, pH 7.4, containing 0.2% triton X-100, 5 mM EDTA, 5 mM EGTA, 2 mM PMSF, 5 mM benzamidine, 2 mM b-mercaptoethanol, and a protease inhibitor cocktail (Sigma-Aldrich, St. Louis, MO, USA).

### Ethics statement

Handling, care and processing of animals were carried out according to regulations approved by the Research Ethics Committee of Hospital de Clínicas de Porto Alegre (Permit Number 12-0229) and the Ethics Committee for Animal Research of the Federal University of Rio Grande do Sul, Porto Alegre, Brazil. All efforts were made to minimize the number of animals used and their suffering.

### High lysine diet and outcome of Gcdh^-/-^ animals under a high Lys (4.7%) intake

Thirty-day-old WT and *Gcdh^-/-^* mice were submitted to a normal chow (containing 20% protein and 0.9% Lys, NUVILAB) or to a high Lys diet (containing 20% protein and 4.7% Lys) for 60 h. After 60 h of diet, the animals were sacrificed by decapitation, the brain was rapidly removed and the cerebral structures dissected. Total RNA was extracted from striatum and cerebral cortex. Most animals under this high Lys chow were asymptomatic although a few (5–10%) *Gcdh^-/-^* mice presented hypotonia and/or moderate paralysis. Only asymptomatic mice were used in the experiments. We also verified in a different set of *Gcdh^-/-^* mice that approximately 20% of them became hypoactive 72 hours after Lys overload and this was followed by paralysis, seizures and death after 5–7 days. These *Gcdh^-/-^* animals under high dietary Lys behaved similarly to those previously described by Zinnanti and collaborators [64].

### RNA extraction and cDNA synthesis

Total RNA was extracted using the RNeasy kit (QIAGEN, Hilden, Germany) according to manufacture's protocol. RNA concentrations were evaluated at 260 and 280 nm absorbances in a NanoDrop 1000 equipment (Thermo Scientific, San Jose, CA, USA). cDNA was synthesized by reverse transcription (RT) reaction using SuperScript III First-Strand Synthesis SuperMix (Invitrogen, Grand Island, NY, USA). The amount of isolated RNA used for RT reactions was 1 µg, 1 µL oligo dT (20), 1 µL of annealing buffer, 10 µL of 2X First-Strand Reaction Mix and 2 µL SuperScript III/RNaseOUT Enzyme Mix in a total reaction volume of 20 µL. Reactions were performed for 5 min at 65°C, 50 min at 50°C and terminated with 5 min at 85°C. Subsequently, cDNA was kept at −20°C until PCR quantification. A 1∶20 dilution of cDNA solution was prepared in water before quantitative real-time polymerase chain reaction (RT-qPCR).

### Quantitative real time PCR (RT-qPCR)

Messenger RNA (mRNA) expression was measured by RT-qPCR using gene-specific TaqMan FAM/MGB inventoried assays (Applied Biosystems, Foster City, CA, EUA) (table 1). Expression values of the targed gene were normalized by the expression of endogenous control beta-actin using TaqMan probe (table 1). Reaction was carried out in a Stratagene MX3000p qPCR System (Stratagene, GE Healthcare Life Sciences, Piscataway, NJ, USA). The cDNA of each mice (n = 5) were used separately in RT-qPCR reaction.

**Table 1:** Number of assays used for relative quantification of each glutamate receptor subunits and transporters (Applied Biosystems).

Receptor	Gene	Assay number
NR1	*Grin1*	Mm00433790_m1
NR2A	*Grin2A*	Mm00433802_m1
NR2B	*Grin2B*	Mm00433820_m1
GluR2	*Gria2*	Mm00442822_m1
GluR6	*GriK2*	Mm00599860_m1
GLT1	*Slc1a2*	Mm00441457_m1
GLAST	*Slc1a3*	Mm00600697_m1
B-actin	*Actb*	Mm00607939_s1

Reactions were carried out in a total volume of 12 µL using 5 µL of cDNA solution, 0.5 µL of gene specific TaqMan assay, 1.5 µL of water milli-Q and 5 µL of Master Mix (Applied Biosystems), containing ROX, Amplitaq Gold DNA polymerase, AmpErase UNG, dATP, dCTP, dGTP, dUTP, and MgCl_2_. Cycling program was 2 min at 50°C, 10 min at 95°C, followed by 40 cycles of 15 s at 95°C and 1 min at 60°C. Reactions were performed in duplicate.

Target transcripts relative expression levels were determined by the ΔΔCt method, according to Livak and Schmittgen [68] using WT mice at each time point as calibrators.

### Western blotting

Brain from 60-day-old WT and *Gcdh^-/-^* mice were submitted to western blotting analysis. Protein extracts (40 µg of total protein) from cerebral cortex and striatum were loaded and eletrophoresed on 10% SDS-PAGE gel, transferred to polyvinylidene fluoride membrane (PVDF), washed in Tris-buffered saline (TBS) and blocked with 5% nonfat dried milk in TBS containing tween 20 (TBS-T). Membranes were then incubated overnight at 4°C with the following primary antibodies: NR2A, NR2B (1∶500 dilution, Millipore, Billerica, MA, USA), GLT1 and GLAST (1∶750, Alpha Diagnostic), and normalized to a loading control (anti-β-actin, 1∶1,000 dilution, Sigma-Aldrich, St. Louis, MO, USA). Membranes were washed extensively with TBS-T solution, incubated for 120 min with HRP-conjugated anti-rabbit IgG (1∶5,000; Millipore), and washed again in TBS-T. Membranes were then immersed in chemiluminescence (ECL) detecting substrate (Millipore, Billerica, MA, USA) for 30 s to 3 min (depending on the antibody), and exposed to radio-X film. Protein bands were quantitatively analyzed with the NIH Image J Analysis software.

### Protein determination

Total protein content was determined by the method of Lowry (1951), using bovine serum albumin as a standard.

### Statistical analysis

Data from mRNA transcripts were expressed as relative quantity that describes the change in expression of the target gene (glutamate receptor and transporter subunits) relative to the reference gene beta-actin. Data from protein expression were expressed as relative density. The results shown in all figures were expressed as mean ± standard deviation (SD) of five separate experiments (animals) performed in duplicate. Comparison between means of WT and Gcdh^-/-^ mice was calculated by the Student's t-test for independent samples. Only significant values are shown in the text. Analyses were performed using the Statistical Package for the Social Sciences software (SPSS, version 14.0). A value of p≤0.05 was considered to be significant.

## Results

### mRNA expression of glutamate receptors in mice fed a normal diet

We initially compared the mRNA expression of subunits of NMDA, AMPA and kainate receptors in the striatum and cerebral cortex from wild type (WT) and *Gcdh* ^-/-^ mice (KO). The mice were fed a normal diet (0.9% lysine) and tested at 7, 30 and 60 days of life.

**Figure 1:**
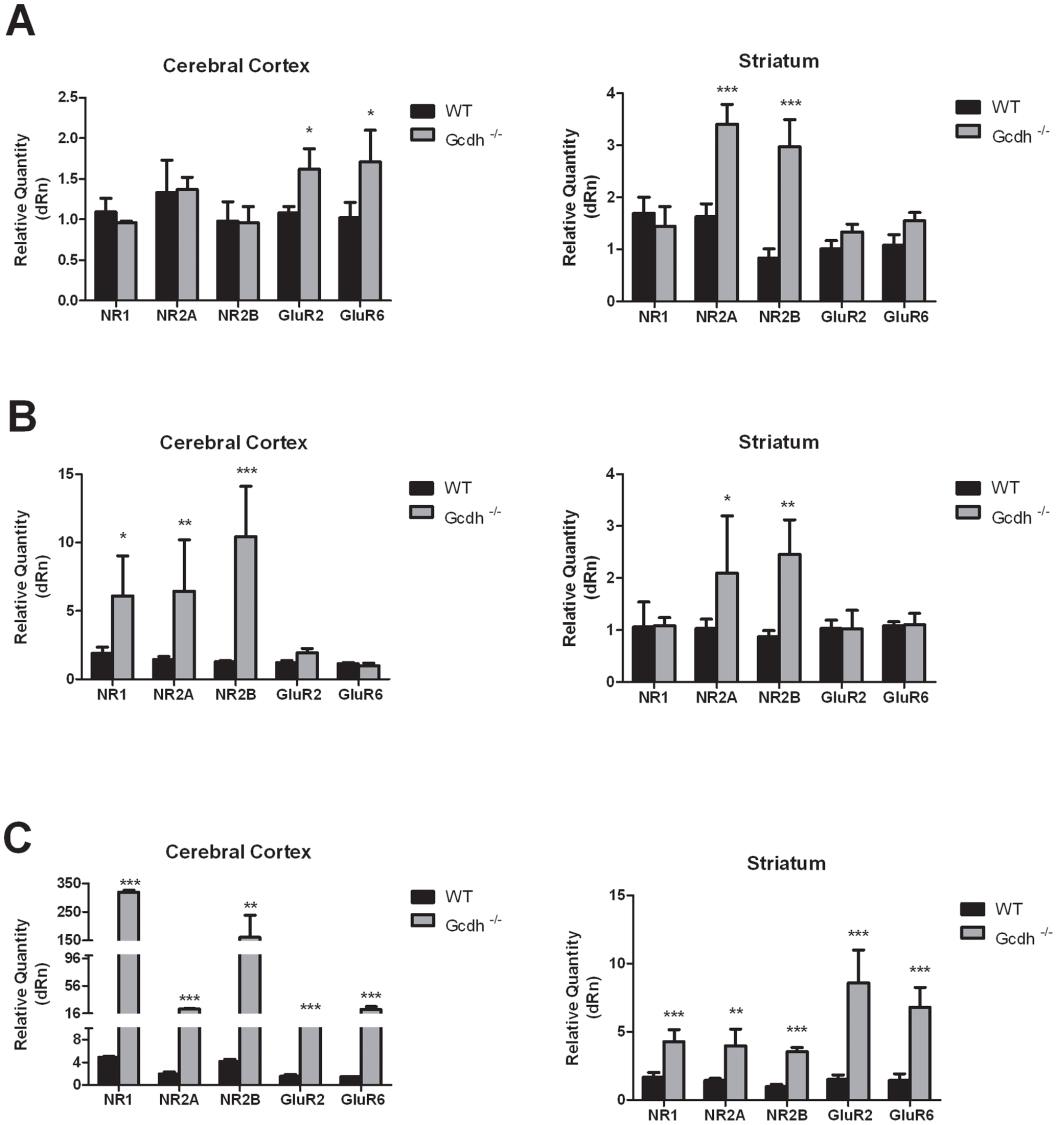
Glutamate receptor mRNA expression levels in cerebral cortex and striatum from wild type (WT) and *Gcdh^-/-^* mice at 7 (A), 30 (B) and 60 (C) days of life. Results are expressed as mean ± standard deviation for five independent experiments (animals) per group. *p≤0.05, **p≤0.01, ***p≤0.001 compared to WT (Student's t test for unpaired samples).

We observed differences in the expression levels of GLUR genes between WT and *Gcdh* ^-/-^ mice in both cerebral cortex and striatum at all ages. mRNA levels of the GluR2 (AMPA) and GluR6 (kainate) subunits were higher (1.4 and 1.7-fold respectively) in cerebral cortex of *Gcdh* ^-/-^ as compared to WT mice at 7 days of life (Figure 1A), whereas in striatum it was the expression of NR2A and NR2B (NMDA) that was much higher (2 to 3.5-fold) (Figure 1A). In contrast, other GLUR subunits showed no differences in mRNA levels between WT and *Gcdh^-/-^* mice at this age.

Furthermore, the expression of NR1, NR2A and NR2B subunits was enhanced 3 to 10-fold in cerebral cortex of 30-day-old *Gcdh* ^-/-^ mice, whereas NR2A and NR2B subunits were also overexpressed (2.0 to 2.5-fold) in striatum (Figure 1B). In contrast, no differences in GluR2 and GluR6 subunits expression between normal and GCDH deficient mice were observed in cerebral cortex and striatum at this age (Figure 1B).

Finally, we demonstrated that all NMDA, AMPA and kainate receptor subunits were significantly more expressed in cerebral cortex and striatum from 60-day-old *Gcdh* ^-/-^ mice (Figure 1C). The figure also shows that the mRNA expression levels of the NMDA receptor subunits NR1, NR2A and NR2B were markedly increased (10 to 80-fold) in cerebral cortex from *Gcdh^-/-^* mice, the same occurring for the GluR2 (AMPA) and GluR6 (kainate) expression but to a lesser degree. Furthermore, all ionotropic receptor subunits were 2 to 5-fold higher in the striatum of *Gcdh^-/-^* mice (Figure 1C).

### mRNA expression of glutamate transporters in mice fed a normal diet

Considering that the glutamate transporter-1 (GLT1) and the glutamate/aspartate transporter (GLAST) are crucial to preventing excitotoxicity by helping to maintain lower concentrations of glutamate in the synaptic cleft, we also evaluated the mRNA expression of these transporters in striatum and cerebral cortex from WT and *Gcdh* ^-/-^ mice.

**Figure 2:**
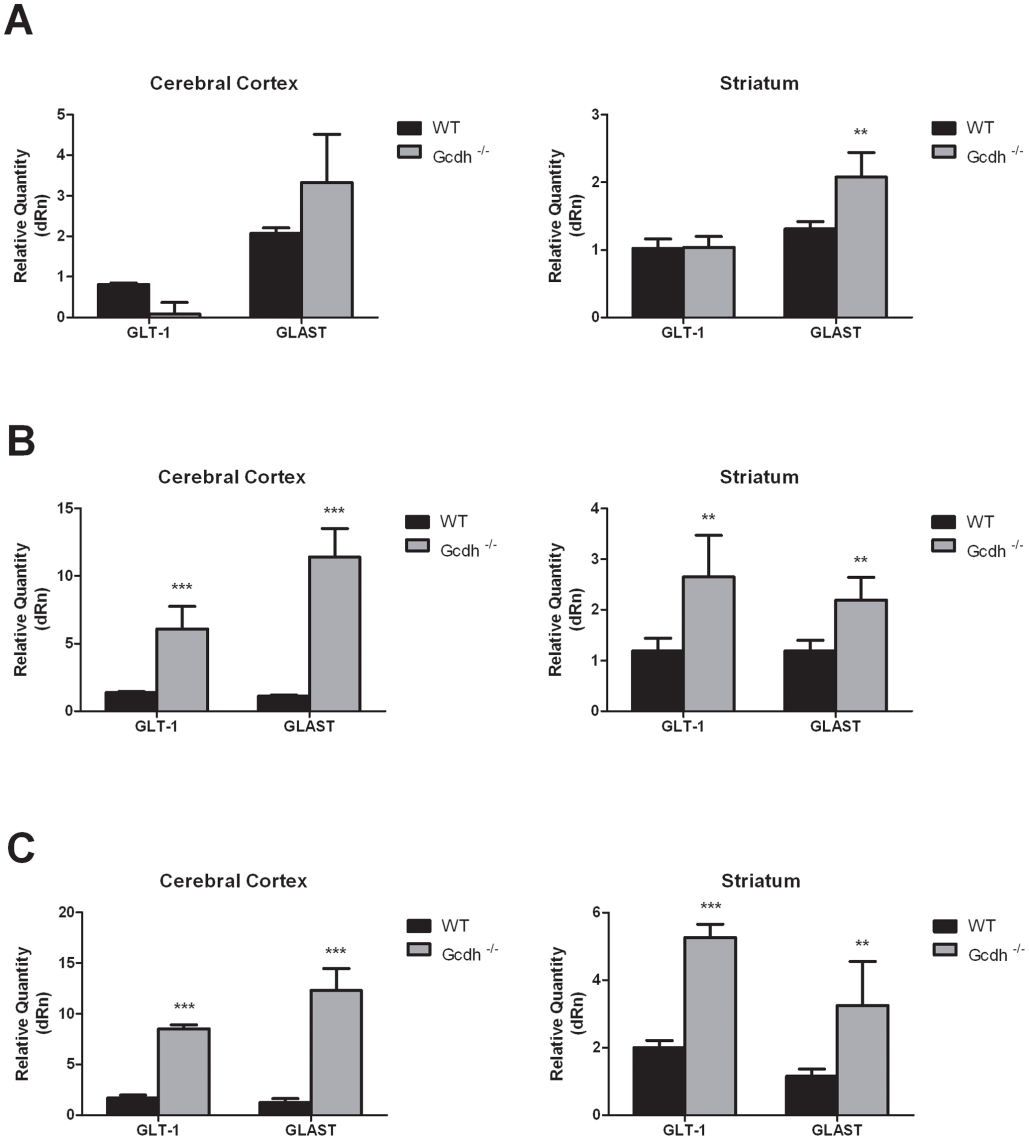
Glutamate transporter mRNA expression levels in cerebral cortex and striatum from wild type (WT) and *Gcdh^-/-^* mice at 7 (A), 30 (B) and 60 (C) days of life. Results are expressed as mean ± standard deviation for five independent experiments (animals) per group. **p≤0.01, ***p≤0.001 compared to WT (Student's t test for unpaired samples).

GLAST expression was significantly higher in striatum but not in cerebral cortex of 7-day-old *Gcdh* ^-/-^ mice (Figure 2A). In contrast, the mRNA levels of GLT1 were not altered in both cerebral structures from *Gcdh* ^-/-^ mice.

In Gcdh^-/-^ mice of 30 and 60 days of life, GLT1 and GLAST mRNA levels were 6 to 12-fold higher in cerebral cortex as compared to age-matched WT mice (Figure 2B and 2C). Similar results were obtained in the striatum, although the differences in expression were lower (2 to 2.5-fold) (Figures 2B and 2C).

### mRNA expression of glutamate receptors and transporters in mice fed a high lysine diet

We also investigated the influence of an enriched lysine diet (4.7%, available for 60 hours) on the expression of glutamate receptors (NMDA, AMPA and kainate) and transporters (GLAST and GLT1) in the cerebral cortex and striatum of 30-day-old WT and *Gcdh^-/-^* mice.

**Figure 3:**
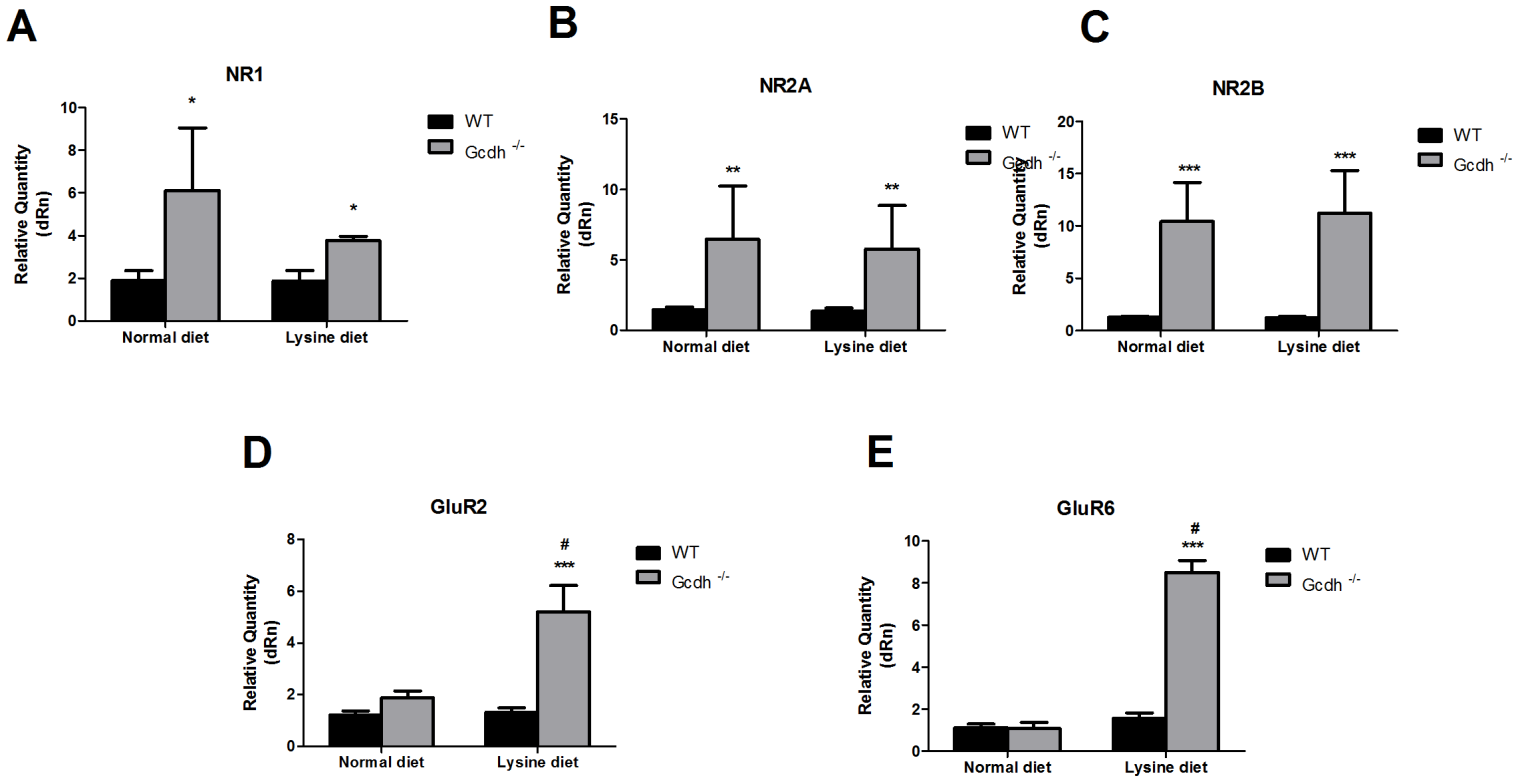
NMDA and non-NMDA receptor mRNA expression in cerebral cortex of 30-day-old wild type (WT) and *Gcdh^-/-^* mice submitted to a normal (0.9% lysine) or high lysine (4.7%) diet. NR1 (A), NR2A (B), NR2B (C), GluR2 (D) and GluR6 (E). Results are expressed as mean ± standard deviation for five independent experiments (animals) per group. *p≤0.05 compared to WT; **p≤0.01, ***p≤0.001 compared to WT; ^#^p≤0.01 compared to *Gcdh^-/-^* mice submitted to a normal diet (Student's t test for unpaired samples).

**Figure 4:**
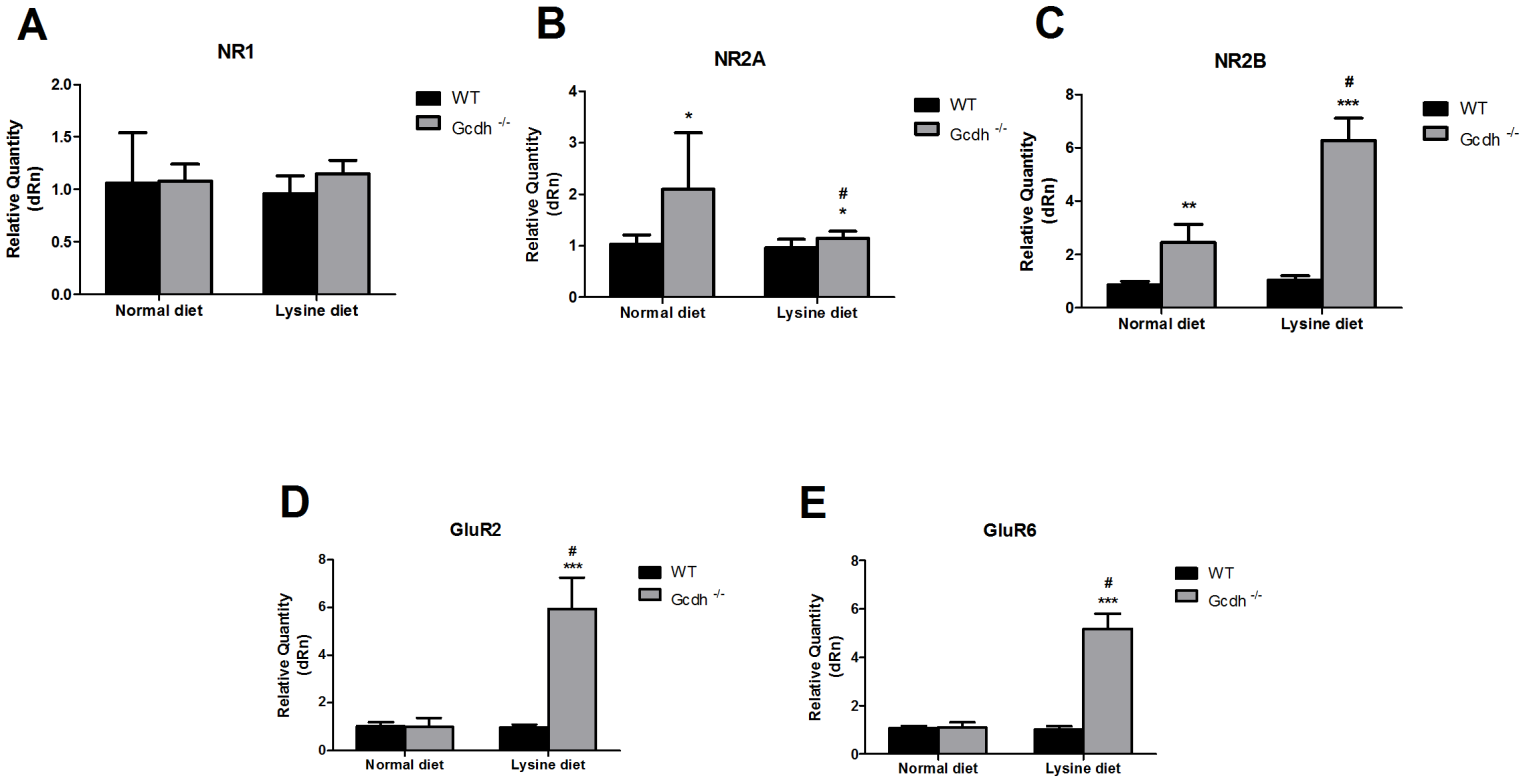
NMDA and non-NMDA receptor mRNA expression in striatum of 30-day-old wild type (WT) and *Gcdh^-/-^* mice submitted to a normal (0.9% lysine) or high lysine (4.7%) diet. NR1 (A), NR2A (B), NR2B (C), GluR2 (D) and GluR6 (E). Results are expressed as mean ± standard deviation for five independent experiments (animals) per group. *p≤0.05 compared to WT; ^#^p≤0.01 compared to *Gcdh* ^-/-^ mice submitted to a normal diet (Student's t test for unpaired samples).

We observed that dietary Lys overload did not alter the differences previously observed in NMDA receptor expression in the cerebral cortex of *Gcdh^-/-^* mice (Figure 3A, 3B and 3C); however, it did augment the expression of particularly the NR2B receptor subunit in the striatum of these genetically modified animals (Figure 4A, 4B and 4C). In addition, there was no significant difference in NR1 mRNA expression between WT and *Gcdh^-/-^* mice in this cerebral structure.

As regards to AMPA (GluR2) and kainate (GluR6) mRNA expression, figure 3D, 3E, 4D and 4E reveals that Lys overload provoked a significantly higher expression of these receptors in both cerebral cortex and striatum of *Gcdh^-/-^* animals. It is of note that a high lysine diet itself did not enhance the expression of these receptors in WT animals, emphasizing the selective action of this diet on the genetically modified mice.

**Figure 5:**
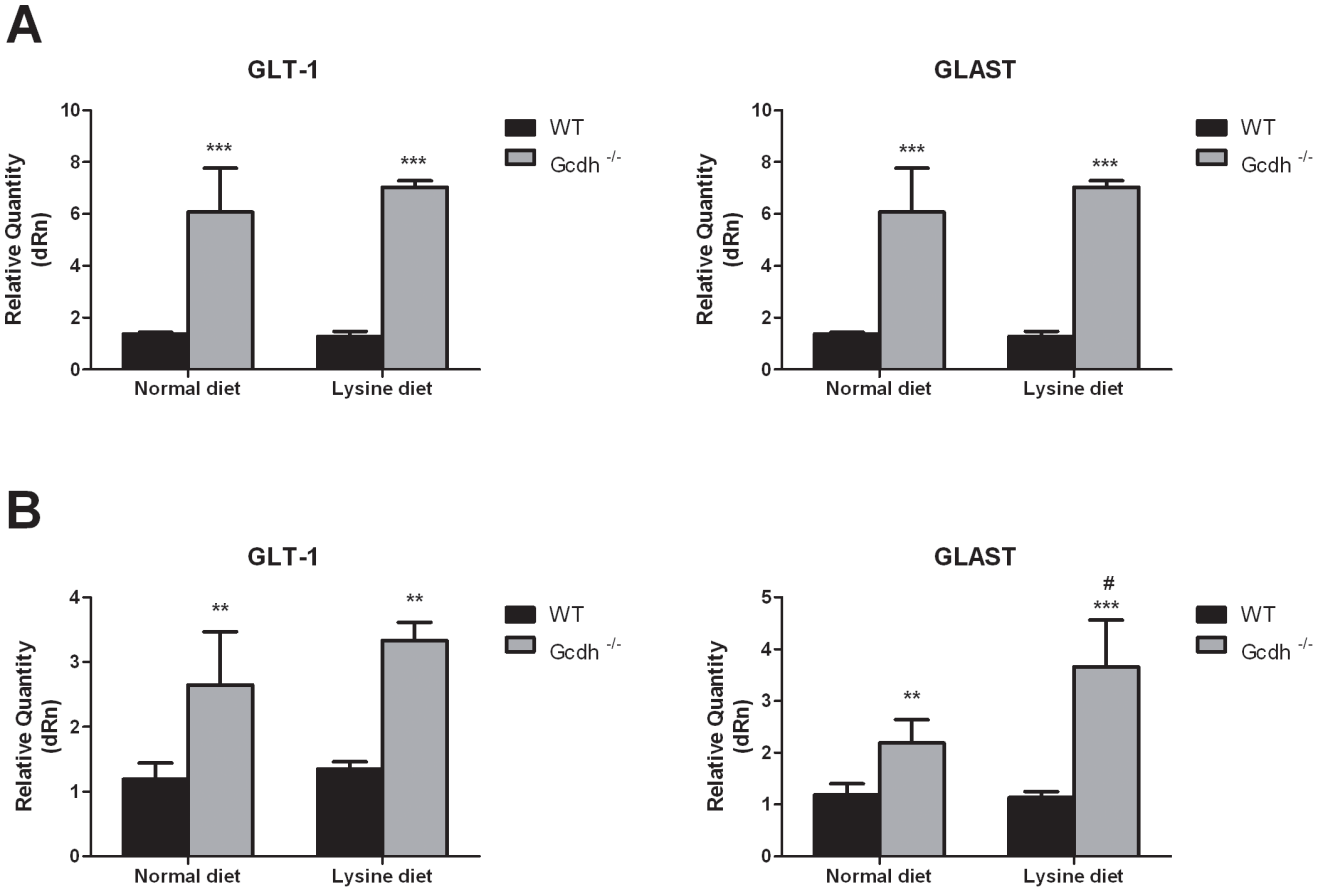
Glutamate transporter (GLT1 and GLAST) mRNA expression in cerebral cortex (A) and striatum (B) of wild type (WT) and *Gcdh-/-* mice submitted to a normal (0.9% lysine) or high lysine (4.7%) diet. A). Results are expressed as mean ± standard deviation for five independent experiments (animals) per group. * p≤0.05, **p≤0.01 compared to WT; #p≤0.05 compared to *Gcdh* ^-/-^ mice submitted to a normal diet (Student's t test for unpaired samples).

With respect to glutamate glial transporters, lysine supplementation did not further enhance the significant differences between WT and *Gcdh* ^-/-^ mice in the expression of GLAST and GLT1 in cerebral cortex (Figure 5A). However, in striatum, GLAST mRNA expression was much increased in *Gcdh* ^-/-^ animals supplied with high lysine (Figure 5B).

### Protein expression levels of glutamate receptors and transporters

We also measured and compared the protein expression of NR2A, NR2B, GLT1 and GLAST by western blotting in cerebral cortex and striatum in *Gcdh* ^-/-^ and WT mice in order to verify whether higher protein levels were also observed in these subunits.

**Figure 6:**
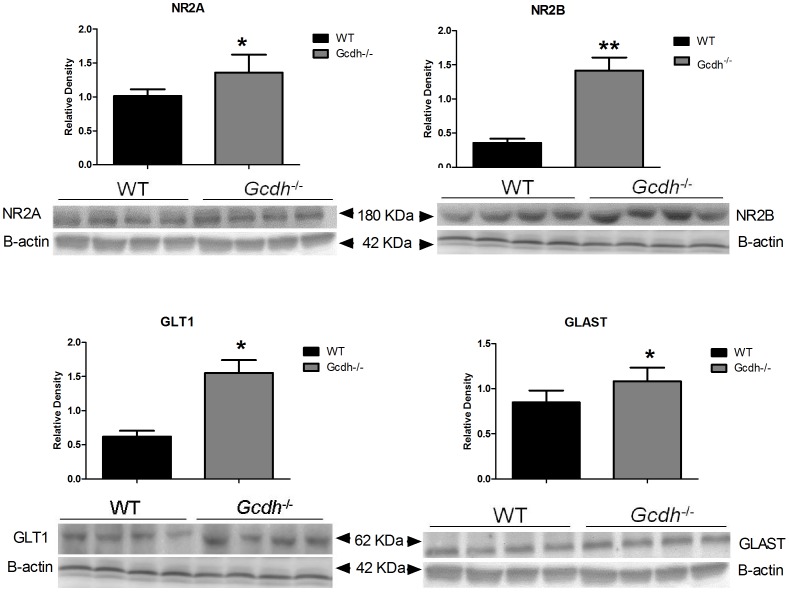
NR2A, NR2B, GLT1 and GLAST protein levels in cerebral cortex of 60-day-old wild type (WT) and *Gcdh* ^-/-^ mice. β-actin was used as the endogenous control. Results are expressed as mean ± standard deviation for four independent experiments (animals) per group. *p≤0.05, **p≤0.01 compared to WT (Student's t test for unpaired samples).

**Figure 7:**
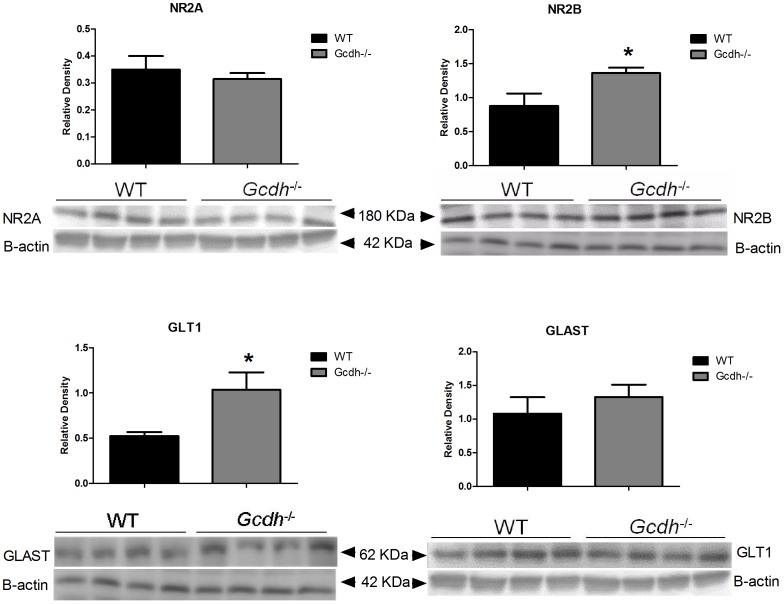
NR2A, NR2B, GLT1 and GLAST protein levels striatum of 60-day-old wild type (WT) and *Gcdh* ^-/-^ mice. β-actin was used as the endogenous control. Results are expressed as mean ± standard deviation for four independent experiments (animals) per group. *p≤0.05 compared to WT (Student's t test for unpaired samples).

We observed higher protein levels of the NR2A (1.3-fold), NR2B (4-fold), GLT1 (3-fold) and GLAST (1.3-fold) in cerebral cortex from *Gcdh* ^-/-^, as compared to WT mice (Figure 6). Regarding to the striatum, there was significant increases of protein levels of NR2B (1.8-fold) and GLT1 in *Gcdh* ^-/-^ mice, with no alteration of NR2A and GLAST protein expression (Figure 7).

## Discussion

We aimed to give new insights on the role of the glutamatergic system on the brain abnormalities observed in patients affected by GA1 [1], [10]–[14]. We therefore determined the mRNA expression of certain subunits of the iGLUR NMDA (NR1, NR2A and NR2B), AMPA (GluR2) and kainate (GluR6) receptors, and of the glial glutamate transporters GLAST and GLT1, and compared their expression between the genetic mice model of this disease (*Gcdh^-/-^*) and WT mice at three ages (7, 30 and 60 days of life). We also investigated the effects of a high lysine diet on the expression of these receptors and transporters in 30-day-old mice.

We observed large differences in expression levels of the genes encoding these receptor subunits and transporter subtypes in cerebral cortex and striatum from *Gcdh^-/-^* mice relative to the WT mice. Overall, *Gcdh^-/-^* mice presented a marked increase of mRNA levels of the GLUR and glutamate transporter subunits, as compared to WT at all ages tested. Furthermore, Lys overload further increased the expression of some but not all receptor and transporter subunits in the striatum and cerebral cortex from *Gcdh^-/-^* mice. Interestingly, Lys overload did not change the expression of these proteins in WT mice.

We initially found that the glutamate receptor subunit NR2B was more expressed (more than 3-fold) in the striatum from *Gcdh^-/-^* mice at 7 days of life. It is of note that the NR2B subunit was previously demonstrated to be involved in excitotoxicity in corticostriatal neuronal cultures supplemented by GA and 3-HGA [30] and is highly expressed in *postmortem* brain of patients with Huntington disease with neuronal degeneration due to glutamate excitotoxicity [69]. Therefore, our findings showing an augmented expression of this subunit in the striatum suggest that this structure may be more susceptible to excitotoxic injury in these animals early in life.

This is in line with other studies showing that brain lesions caused by NMDA agonists are more pronounced in striatum than in other brain regions from 7-day-old rats, implying that excitotoxicity is extremely active in the immature brain, especially in the striatum [70]. Accordingly, the increased expression of specific NMDA receptor subunits during rat development (7–14 days) parallels the higher susceptibility of the CNS to neurotoxins targeting the NMDA receptor system [66], [71]. Interestingly, the pattern of NMDA-subunit expression seems to be similar in rat and humans, with ubiquitous NR1 subunit expression throughout development and adulthood, and high levels of NR2B early in development, decreasing afterwards, whereas subunit expression NR2A increases into adulthood [72]–[74].

We also observed that at 7 days of life the mRNA expression of AMPA and kainate receptors was higher in cerebral cortex from *Gcdh^-/-^* relative to WT mice. Although we cannot at the present establish the significance of these data, it should be emphasized that the kainate receptor GLUR6 subunit seem to play an important role in ischemia-induced JNK3 activation and neuronal cell death [75], being therefore cytotoxic. On the other hand, the functional GluR2 subunit receptor is of interest because of its ability to “shut down” calcium influx through AMPA receptors [76], [77], thereby protecting cells from excitotoxicity. Considering the role of this AMPA receptor subunit making neurons less permeable to calcium, the higher expression of the GluR2 subunit in *Gcdh^-/-^* mice may be viewed as a mechanism for the protection of cortical neurons from the toxic effects of glutamate in early life. Taking these observations together, we cannot at present predict the consequences of the combined opposite effects of high expression of GluR2 and GluR6 in the immature cerebral cortex, as found in this study.

At 30 days of life, the mRNA expression of the NMDA receptor NR2A and NR2B subunits was higher in the cerebral cortex and the striatum from the *Gcdh^-/-^* animals receiving a normal diet; and Lys overload caused a more accentuated increase of these NMDA receptor subunits only in the striatum. Although no difference was observed in the expression of the AMPA and kainate subunits in the *Gcdh^-/-^* animals fed a normal diet, high dietary lysine promoted significantly higher expression of these non-NMDA receptors in both cerebral structures as compared to WT mice. Interestingly, these NMDA and non-NMDA subunits were not increased in WT receiving a high Lys overload, implying that increased transcription of these subunits in *Gcdh^-/-^* mice was not due to Lys itself, but probably due to one or more of its by-products, including GA and 3-HGA. Considering that it has been previously demonstrated that a similar enriched Lys diet (4.7% Lys) induced striatal and cortical lesions, as well as mitochondrial biochemical alterations in 4-week-old *Gcdh^-/-^* mice [65], it may be tentatively presumed that the higher expression of GLURs might be involved in these findings.

We also demonstrated that the mRNA levels of all GLUR subunits (NMDA and non-NMDA) were markedly elevated especially in cerebral cortex but also in striatum from 60-day-old *Gcdh^-/-^* animals, probably suggesting that at this older age these cerebral structures, and particularly the cerebral cortex from the GCDH deficient mice, are more susceptible to glutamate toxicity. Prior studies have shown that following a high Lys diet 4-week old *Gcdh^-/-^* mice suffer severe brain injury and death, whereas most 8-week-old (approximate 60 days of life) *Gcdh^-/-^* mice survive up to 6 weeks on a high Lys diet, eventually developing white matter lesions along with neuronal loss and increased numbers of reactive astrocytes [64], [65]. Survival of 8-week-old mice to long-term exposure to a high Lys diet was associated with a decreased accumulation of brain GA as compared to the 4-week-old animals, which the authors suggest may be related to a decreased permeability of the blood brain barrier to Lys in the older mice [65]. The development of striatal injury in the older *Gcdh^-/-^* mice after long-term Lys exposure, in spite of decreased accumulation of GA, supports a potential role for the increased GLUR expression we observed in 60-day-old animals in mediating susceptibility to Lys toxicity.

As there is usually only moderate correlation between mRNA and protein levels [78], we also measured the protein expression of NR2A, NR2B, GLT1 and GLAST by western blotting in cerebral cortex and striatum in order to verify whether the higher transcripts of glutamate receptors and transporters were translated into their products. We chose the glutamate receptor NR2A and NR2B because mRNA expression of these subunits was highly enhanced in *Gcdh^-/-^* mice, besides being important for the functional properties of NMDA receptors [79], [80]. Furthermore, the involvement of NR2B was previously postulated to be involved in excitotoxicity in GA I [30], [81].

We found that the protein levels of all examined glutamate receptor subunits (NR2A and NR2B) and transporter subtypes (GLT1 and GLAST) were significantly increased in cerebral cortex from 60-day-old *Gcdh* ^-/-^, as compared to the WT mice, supporting a higher expression of these proteins in the genetic mice model of GA I. In striatum, the protein levels of the NR2B receptor subunit and of the GLT1 transporter subtype were similarly increased in *Gcdh* ^-/-^, mice, with no statistical difference for NR2A and GLAST proteins. Furthermore, the magnitude of the differences in protein levels was lower relatively to the variation observed in their mRNA expression. This may be due to the fact that proteins are under a complex posttranslational regulation [46]. In this context, increased neural death or protein degradation due to protein instability, or changes in receptor and transporter assembly and presentation at the cell surface may possibly explain our present findings [46].

Although we do not know the molecular mechanisms underlying the marked overexpression of glutamate receptors found in the present study, it may be secondary to the overactivation of these receptors ultimately leading to gene activation [46]. In case that activation of GLURs is the primary signal, possible candidates for reacting with these membrane proteins include glutamate itself and/or possibly GA and 3-HGA, which are found at higher amounts in the brain of the *Gcdh^-/-^* mice and are structurally similar to glutamate. In fact, there is experimental evidence that these organic acids interfere with glutamate receptors and transporters. GA stimulates glutamate binding to receptors and glutamate uptake into astrocytes and inhibits vesicular and synaptosomal glutamate uptake [34]. There is also some evidence showing that 3-HGA interacts with NMDA receptors, provokes a significant calcium uptake by cortical slices [33] and enhances glutamate uptake into astrocytes [35]. These data are in agreement with older studies demonstrating that GA and particularly 3-HGA are excitotoxic towards cultured neurons [27], [31], [36], [82]–[84].

The present data are consistent with the hypothesis of a higher susceptibility of distinct regions of the brain to excitotoxic injury mediated by overactivation of specific iGLUR subunits [43], [66], [67]. This is also in line with the great deal of evidence showing that overstimulation of glutamate receptors has been associated with neurodegeneration in various clinical disorders in the developing brain, such as hypoxia-ischemia, epilepsy, physical trauma and some genetic abnormalities of amino acid metabolism [85]–[90].

Glutamate transporters may affect the sensitivity of brain to excitotoxic insult. We observed that in 7-day-old *Gcdh^-/-^* mice only striatal GLAST mRNA expression was significantly higher, with no alterations of GLT1 in striatum or cerebral cortex. Moreover, GLAST expression increased even more in the striatum of *Gcdh^-/-^* mice submitted to a high dietary Lys intake. It is of interest that GLAST transporter is responsible for most glutamate uptake from the synaptic cleft in the immature brain, being expressed primarily at early stages of development [52], [53]. At postnatal days 30 and 60 both GLAST and GLT1 were more expressed in cerebral cortex and striatum from *Gcdh^-/-^* mice. Taken together, these data suggest that a higher expression of glutamate transporters, especially GLAST at early stages of development, may be necessary to regulate glutamate levels in the synaptic cleft, therefore avoiding excitotoxicty [41].

In this context, it has been previously shown that glutamate and other agonists of glutamate transporters may induce the expression of these astrocytic proteins, and particularly GLAST [91], [92]. Increased glutamate, and possibly GA or 3-HGA, concentrations in the synaptic cleft may induce a higher expression of these transporters in the *Gcdh^-/-^* mice. It also should be considered that higher expression and activation of iGLURs in the presynaptic neuron would increase calcium influx secondarily leading to glutamate release into the synaptic cleft, increasing, therefore, the concentration of glutamate in this space. Therefore, it can be presumed that the higher expression of GLURs may be accompanied by a greater transcription of glutamate transporters to reduce glutamate extracellular concentrations.

Furthermore, GA and 3-HGA may function as agonists of glutamate receptors and/or transporters, secondarily inducing a higher expression of glutamate transporters [32], [34], [36], [82], [93], [94]. On the other hand, it should be considered that glutamate uptake into astrocytes is Na^+^-dependent and coupled with the activation of Na^+^,K^+^-ATPase to remove the excess of sodium from the cytoplasm, and this process depends on a large ATP supply, utilizing about 50% of ATP produced in the brain [95]–[97]. We recently demonstrated a marked reduction of this enzyme activity in the brain of *Gcdh^-/-^* mice [18], [19] that may be due to the directly inhibitory effects of GA on this activity [82], [98] or to an impairment of brain energy homeostasis [65]. Therefore, we cannot rule out that a low activity of the enzyme Na^+^,K^+^-ATPase that is necessary for glutamate uptake into astrocytes may also lead to increased extracellular levels of glutamate in the *Gcdh^-/-^* mice model.

The level of expressed glutamate receptors and transporters reflects a balance between transcription, translation, mRNA level, protein stability, receptor assembly, and presentation at the cell surface, all of which are integrated through numerous environmental stimuli. Our data clearly show a very high mRNA expression of glutamate receptors and transporters and a greater but less intense protein expression of some of these membrane surface proteins in the *Gcdh^-/-^* mice.

In summary, the current study is the first to investigate the mRNA and protein expression of iGLUR subunits and of glutamate transporter subtypes in *Gcdh^-/-^* mice at three distinct postnatal ages in two cerebral structures that are most damaged in GA I. We demonstrated a regional-specific higher expression of various iGLUR subunits in the cerebral cortex and striatum of *Gcdh^-/-^* mice. We also showed that high Lys overload leads to a more prominent expression of various subunits of GLURs and transporters in these animals, which may underlie the vulnerability of the *Gcdh^-/-^* animals to Lys-induced brain injury [64]. The present results suggest the possibility that disturbances of glutamatergic neurotransmission may play a role in GA I pathophysiology. The neuropathological abnormalities found in postmortem examination of the basal ganglia and cerebral cortex of patients with GA I are in line with our findings, which include postsynaptic vacuolization characteristic of glutamate-mediated brain damage [1], [99].
